# Editorial Comment: Use of indocyanine green angiography in microsurgical subinguinal varicocelectomy - lessons learned from our initial experience

**DOI:** 10.1590/S1677-5538.IBJU.2017.0107.1

**Published:** 2017

**Authors:** Marcello Cocuzza

**Affiliations:** 1 Departamento de Urologia, Universidade de São Paulo, SP, Brasil

Use of indocyanine green angiography in microsurgical subinguinal varicocelectomy - lessons learned from our initial experience.

Dr. Chak-Lam et al., from China, produce an elegant manuscript that describe the use of intraoperative indocyanine green angiography with infrared fluorescence operative microscope in microsurgical subinguinal varicocelectomy that potentially allows an early identification as well as preservation of testicular artery (1).

The main goal of varicocelectomy is to preserve testicular function and initiate pregnancy in infertile couples (2). Although surgical repair of a varicocele can be performed using several methods, microsurgical repair appears to be associated with better outcomes and lower complication rates (3). The most common complications associated with conventional approaches include postoperative varicocele recurrence or persistence, hydrocele formation and testicular artery injury (4).

Most infertility experts prefer the subinguinal approach that is performed through an incision below the external inguinal ring obviating the need to open the aponeurosis of the external oblique which causes less pain (5). However, the number of spermatic arteries identified in the spermatic cord seems to be dependent on the level of the dissection. The augmented probability of encountering multiple spermatic arterial branches in the subinguinal region contributes to the technical difficulty of this approach (5). Therefore, the use of an operating microscope providing magnification up to 25x allows a better preservation of the testicular arteries and lymphatic vessels during subinguinal approach.

Previous studies reported that multiple spermatic arteries are identified in approximately 40% of the spermatic cords during microsurgical varicocelectomy at the subinguinal level (5, 6). Jarow et al., found numerous arterial branches in 81% of the spermatic cords upon histological analysis, which is higher than clinical observation studies (7). Therefore, it is possible that an inadvertent unrecognized ligation of a small (secondary) internal spermatic artery occurs more frequently than reported. Although there is not unanimity about the necessity to preserve all testicular arterial branches, this might be responsible for suboptimal spermatogenic recovery or failure to improve fertility in some cases (4). Data from our group published in 2010, showed that systematic use of intraoperative Doppler during subinguinal microsurgical varicocele repair allows a higher number of arterial branches preserved as well as a superior number of internal spermatic veins ligated (8). A solitary artery was identified in 45.5% of cords, while 2 arteries were identified in 43.5% and 3 or more arteries were identified in 11%. The identification of the main spermatic artery can be confirmed by visualization of clear pulsatile movement; however, the identification of tiny secondary arteries is not always so evident.

In the present study, the authors found a low average number of arteries using intraoperative indocyanine green angiography compared to published data mentioned above. These findings could be explained by an aggressive manipulation of the vessels during dissection that can lead to spasm, making it difficult to achieve the ideal indocyanine green perfusion especially in secondary tiny arteries. In addition, the present study has some limitations that must be addressed before we can conclude that a combination of subinguinal microsurgical varicocelectomy and indocyanine green angiography may decrease the incidence of artery injury during varicocele repair. For example, the authors did not compare the benefits of this new technique with conventional microsurgical procedures. Also, the number of patients studied is too small to draw definite conclusions. However, the authors must keep in mind that current results highlight how the continued investigation appears worthwhile and additional research is needed to better clarify whether all these technological advances is likely to improve testicular function, seminal parameters or even fertility potential.

In conclusion, the testicular artery is the largest artery supplying the testicle and its ligation during varicocele repair may exert deleterious effects on testicular function. Thus, there is growing support for artery sparing techniques and accidental arterial injury during varicocelectomy should be avoided with all effort possible. Current available data indicates that preoperative parameters are not predictive of the number of testicular arteries identified at the time of surgery. As a result, the application of technological advances during varicocelectomy including optical magnification, microsurgery skills, vascular Doppler and indocyanine green angiography should be alternative options to achieve maximal preservation of the arterial blood supply to the testes.
